# Advances in the role of resveratrol and its mechanism of action in common gynecological tumors

**DOI:** 10.3389/fphar.2024.1417532

**Published:** 2024-07-17

**Authors:** Qian Yang, Dandan Meng, Qingchen Zhang, Jin Wang

**Affiliations:** ^1^ College of Chinese Medicine, Shandong University of Traditional Chinese Medicine, Jinan, China; ^2^ First Clinical Medical College, Shandong University of Traditional Chinese Medicine, Jinan, China

**Keywords:** resveratrol, gynecological tumors, malignant tumors, antitumor mechanisms, apoptosis

## Abstract

The incidence of common gynecological malignancies remains high, with current treatments facing multiple limitations and adverse effects. Thus, continuing the search for safe and effective oncologic treatment strategies continues. Resveratrol (RES), a natural non-flavonoid polyphenolic compound, is widely found in various plants and fruits, such as grapes, Reynoutria japonica Houtt., peanuts, and berries. RES possesses diverse biological properties, including neuroprotective, antitumor, anti-inflammatory, and osteoporosis inhibition effects. Notably, RES is broadly applicable in antitumor therapy, particularly for treating gynecological tumors (cervical, endometrial, and ovarian carcinomas). RES exerts antitumor effects by promoting tumor cell apoptosis, inhibiting cell proliferation, invasion, and metastasis, regulating tumor cell autophagy, and enhancing the efficacy of antitumor drugs while minimizing their toxic side effects. However, comprehensive reviews on the role of RES in combating gynecological tumors and its mechanisms of action are lacking. This review aims to fill this gap by examining the RES antitumor mechanisms of action in gynecological tumors, providing valuable insights for clinical treatment.

## 1 Introduction

The incidence of gynecological malignant tumors, a major cause of female mortality worldwide, remains high and poses a significant threat to women’s health (Sung et al., 2021). The most common gynecological tumors include cervical carcinoma (CC), endometrial carcinoma (EC), and ovarian carcinoma (OC). Studies have shown that both genetic mutations and epigenetic alterations are critical factors in promoting cancer development and progression (Tomasetti et al., 2017; Yang et al., 2021). However, the pathogenesis of these cancers is not yet fully understood, resulting in a lack of effective therapeutic strategies. Despite significant advances in treating common gynecological tumors, current first-line regimens, including radiotherapy, chemotherapy, and surgery, have multiple limitations and adverse effects. Chemotherapeutic drugs, although broadly effective against a wide range of tumors, often harm normal tissues due to their inability to precisely target tumor cells, leading to significant side effects. In recent years, tumor immunotherapy and targeted therapy have shown great potential (Jin et al., 2019; Xiang et al., 2021; Zhao et al., 2021; Wang et al., 2022). Although these therapies have yielded some positive results compared to conventional treatments, they have not fundamentally improved the problems of adverse effects and patient quality of life. Furthermore, advanced gynecological tumors are prone to metastasis and recurrence, rendering prevention and treatment challenging. Therefore, exploring safe and effective tumor treatment strategies remains a critical priority in oncology research.

**FIGURE 1:**
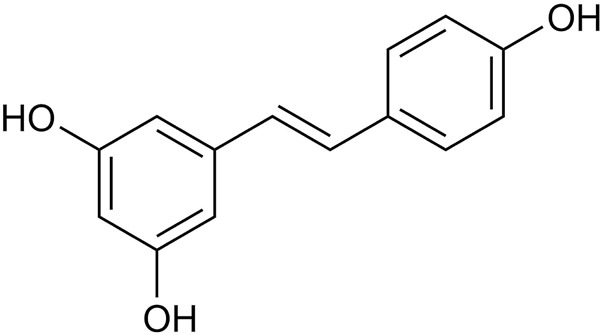
Chemical structure of resveratrol.

Natural botanicals show significant potential in preventing and treating various types of cancer. These botanicals not only have synergistic anti-tumor effects but also improve patients’ quality of life by reducing side effects during radiation and chemotherapy (Moody et al., 2020). Notably, they have demonstrated therapeutic potential for a wide range of cancer types, primarily due to their ability to modulate pathways involved in cancer onset and progression (Hsu and Hwang, 2019; Wang et al., 2020a; Saggam et al., 2020). Resveratrol (RES), a natural non-flavonoid polyphenolic compound, is widely found in various plants and fruits, particularly in *P. suffruticosa Andr. Var. Papaveracea (Andr.) Kerner* and *Reynoutria japonica Houtt.* (Tian and Liu, 2020). RES is a major component in the roots of the medicinal plant *R. japonica Houtt.* (Hou et al., 2024). Furthermore, the Compendium of Materia Medica records that *R. japonica Houtt.* Has a variety of effects, such as invigorating blood circulation, removing blood stasis, clearing heat, and draining pus (Li, 2014). RES (*trans*-3, 4, 5-trihydroxystilbene) (Figure 1), with the molecular formula C_14_H_12_O_3_, appears as a white to yellowish powder in its pure form. Due to the presence of two phenolic rings interconnected by a bis-styrene bond, RES exists in both *trans*- and *cis*-isomeric forms. Among these, the *cis* isomer is not suitable for commercial applications due to its instability. However, the *trans* isomer of RES exhibits higher stability and greater biological activity than the *cis* isomer (Wenzel and Somoza, 2005).

RES exhibits a wide range of biological activities, including antioxidant (Iuga et al., 2012; Brenjian et al., 2020), anti-inflammatory (Alrafas et al., 2020; He et al., 2021), antitumor (Khusbu et al., 2020), cardiovascular protection (Gal et al., 2021), neuroprotection (Abd Aziz et al., 2020), hypoglycemic (Joycharat et al., 2018), and antiviral effects. (Zhao et al., 2018). These multi-faceted biological activities render RES useful in the treatment and prevention of diseases, such as cardiovascular disease, Alzheimer’s disease, diabetes, and cancer. RES influences all stages of carcinogenesis (tumor initiation and promotion) and progression (angiogenesis and metastasis) (Ren et al., 2021). Notably, RES demonstrates a broad spectrum of antitumor properties and exhibits significant inhibitory effects on various cancer cell types, including lung (Li et al., 2020), renal cell carcinoma (Tian et al., 2020), hepatocellular carcinoma (Zhang et al., 2020), and ovarian cancer (Vergara et al., 2012). The mechanisms of action of RES involve inducing tumor cell apoptosis via multiple pathways, inhibiting proliferation, migration, and invasion, and enhancing the drug efficacy while reducing toxicity when used in combination with chemotherapy or targeted drugs. RES exerts its anticancer functions by regulating signaling pathways, such as Wnt/β-catenin, Hedgehog, mitogen-activated protein kinase (MAPK), and nuclear factor kappa B (NF-κB) (Xie et al., 2023; Ye et al., 2023). In this review, we examine the anticancer mechanisms and targets of RES in common gynecological tumors, aiming to provide a new theoretical basis for treatment.

## 2 RES background, absorption, and metabolism

RES was first discovered and isolated in 1940 from the roots of *Veratrum grandiflorum* (Cione et al., 2019). Subsequent studies revealed that RES is widely found in various common medicinal plants, such as *R. japonica Houtt.*, *C. tora L.*, *M. alba L.*, and *F. multiflora (Thunb.) Harald.* Furthermore, RES has been isolated from crops, such as grapes and peanuts (Tian and Liu, 2020). RES plays a crucial defense role in plants as an antitoxin; for example, its synthesis significantly increases when plants are attacked by pathogenic bacteria or are placed in unfavorable environments (Malaguarnera, 2019). Moreover, RES is regarded as a phytoestrogen (Gehm et al., 1997). Since the anticancer effects of RES were first reported in 1997, its mechanism of action against a wide range of cancer cells has been extensively studied.

The clinical application of RES is limited due to its poor water solubility, short half-life, rapid absorption and metabolism by the body, and low bioavailability (Huang et al., 2019). The oral absorption of RES is approximately 75% (Walle, 2011). After oral ingestion, RES can be absorbed in large quantities by intestinal cells via passive diffusion or carrier-mediated transport across the apical cell membrane. It is then rapidly converted by phase II enzymes in the liver, forming mainly glucuronide or sulfate, which is subsequently excreted by the body through the urine (Lançon et al., 2004; Ratz-Łyko and Arct, 2019). Consequently, relatively low levels of unmetabolized RES are available in the systemic circulation after oral ingestion (Boocock et al., 2007a). Furthermore, RES exhibits a high volume of distribution in the human body due to its lipophilic nature (Boocock et al., 2007b; la Porte et al., 2010). Animal experiments have shown that upon ingestion, RES is concentrated in organs with abundant blood flow, such as the liver, heart, kidneys, and brain (Liang et al., 2013; Menet et al., 2017). However, due to the difficulty in obtaining biopsy samples, little is known about the detailed distribution of RES in different human tissues of the human body, and relevant research information is lacking (Juan et al., 2010). Human oral metabolism assays for RES have indicated that the peak drug mass concentration of the RES prototype was <10 ng/mL, and the plasma concentration of RES with total metabolites was only 400 ng/mL at 0.5–2.0 h following a single oral dose of 25 mg (Goldberg et al., 2003; Walle et al., 2004). Additionally, Walle et al. observed a significant second peak in total plasma radioactivity levels 6 h after oral RES administration (25 mg dose) (Walle et al., 2004), hypothesizing that this may be due to the reabsorption of the conjugated metabolite into the intestine, resulting in an intestinal recirculation effect. In a similar study by Brown et al., the dose-dependent pharmacokinetics of multiple administrations were evaluated over a 29-d period, showing a significant elevation in RES blood concentrations in participants after continuous administration of 5 g doses (Brown et al., 2010).

## 3 Role of RES in inducing tumor cell apoptosis

Apoptosis is a key component in maintaining tissue homeostasis (Morana et al., 2022). As apoptosis inhibition is a characteristic of tumor cells (Pistritto et al., 2016), inducing apoptosis is an important strategy in tumor therapy.

### 3.1 B-cell lymphoma-2 (Bcl-2) family of molecules

The Bcl-2 family coordinates apoptosis and regulates mitochondrial outer membrane permeability. Bcl-2 proteins block apoptosis in tumor cells (Fitzsimmons et al., 2020), whereas Bcl-2-associated X protein (Bax) promotes apoptosis (Jeng et al., 2018). Li et al. (2018) reported that RES treatment increases Bax expression and decreases Bcl-2 and B-cell lymphoma-extra-large (Bcl-XL) expression, leading to increased apoptosis in CC (HeLa) cells. RES induces apoptosis in OC A2780 cells by increasing Bax protein expression and activating caspase-3 in normoxic environments (Synowiec et al., 2023). Yao et al. (2021) found that RES treatment effectively accelerates apoptosis in OC cells (SKOV-3 and OV-90) by increasing miR-34a levels and decreasing Bcl-2 levels, suggesting that its pro-apoptotic mechanism might involve the miR-34a/Bcl-2 axis. Tang et al. (2015) reported that RES induces apoptosis in OC cells by upregulating Bax and caspase-3 expression and downregulating Bcl-2 expression in the human OC cell line, SKOV-3.

### 3.2 p53 oncogenes

p53 is a critical oncogene in the human body, and the loss of its function is a prerequisite for cancer development (Zhang et al., 2020). RES promotes apoptosis in CC cell lines (HeLa and CaSki) (Sun et al., 2021) by inhibiting the expression of the human papillomavirus E6 and E7 proteins (E6/E7) and significantly increasing p53 and Bax levels, which promote G1/S phase blocking. This finding is consistent with that of Chatterjee et al. (2018), who reported that RES treatment suppresses E6 expression in CC cells (HeLa), significantly enhancing p53-mediated apoptosis. Moreover, Flores-Pérez and Elizondo (2018) also found that RES promotes apoptosis in HeLa cells. Specifically, RES promotes G1/S phase cell blockade and induces apoptosis by increasing p53 expression and inducing G1/S phase cell blockade.

### 3.3 Endoplasmic reticulum (ER) stress

Proper ER function, particularly in protein synthesis, folding, and modification, is vital for cell survival (Schwarz and Blower, 2016). Disrupting homeostasis triggers the accumulation of unfolded or misfolded proteins in the ER, inducing an unfolded protein response (UPR) (Di Conza and Ho, 2020). A mild UPR maintains cellular homeostasis, whereas a sustained, overly strong UPR induces apoptosis (Koksal et al., 2021). Gwak et al. (2016) studied OC cells and showed that by inhibiting protein kinase B (AKT) activation, RES triggers glycogen synthase kinase-3β (GSK3β) activation and downregulates ectonucleoside triphosphate diphosphohydrolase 5 (ENTPD5) expression, ultimately interfering with N-linked protein glycosylation and inducing ER stress-mediated apoptosis.

### 3.4 Other mechanisms of RES-induced apoptosis in tumor cells

**FIGURE 2:**
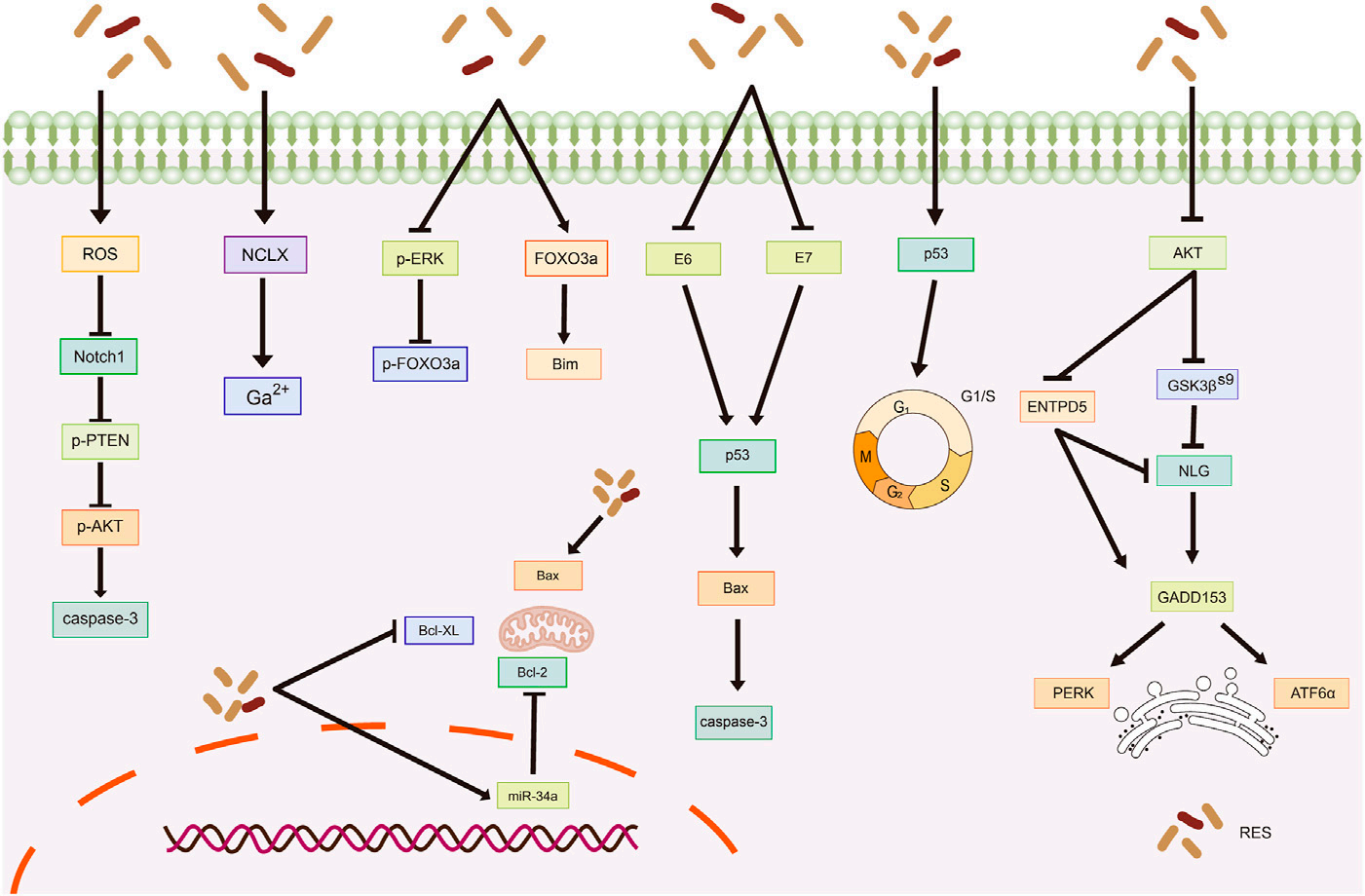
Mechanism of resveratrol inducing apoptosis in common gynecological tumors. RES, resveratrol; ROS, reactive oxygen species; Notch1, Neurogenic locus notch homolog protein 1; p-PTEN, phospho- Phosphatase and tensin homolog; p-AKT, phospho-Protein kinase B; NCLX, sodium/lithium/calcium exchanger; FOXO3a, Forkhead box O3; Bim, Bim Protein; E6, Human papillomavirus E6 protein; E7, Human papillomavirus E7 protein; Bcl-2, B-cell lymphoma-2; Bax, Bcl-2-associated X; Bovine B-cell lymphoma-XL; GSK3β(S9), glycogen synthase kinase-3β(Ser9); ENTPD5, ER UDPase, ectonucleoside triphosphate diphosphohydrolase 5; NLG, N-linked glycosylation; GADD153, rowth arrest/DNA damage-inducible protein153; PERK, Protein Kinase RNA-like Endoplasmic Reticulum Kinase; ATF6α, Recombinant Activating Transcription Factor 6-α.

The sodium/lithium/calcium exchanger (NCLX) is a key protein involved in maintaining intracellular calcium homeostasis (Takeuchi et al., 2020; Britti et al., 2021). Devi et al. (2021) reported that NCLX mRNA levels were significantly upregulated in HeLa cells after RES treatment, leading to increased intracytoplasmic calcium ions and ultimately triggering calcium homeostasis disruption-mediated apoptosis. In another study, RES treatment inhibited extracellular signal-regulated kinase (ERK) and Forkhead box O (FOXO) 3a phosphorylation while increasing FOXO3a and Bim expression and promoting FOXO3a nuclear translocation, which induced apoptosis in HeLa cells (Liu et al., 2020). Reactive oxygen species (ROS) are highly reactive, short-lived chemically active molecules. Tumor cell proliferation often coincides with elevated ROS production (Hayes et al., 2020). ROS induces apoptosis in tumor cells by mediating oxidative stress (Moloney and Cotter, 2018; Srinivas et al., 2019). According to Kim et al. (Kim et al., 2019), RES treatment stimulates ROS generation in cells, which inhibits neurogenic locus notch homolog protein 1 (Notch1) signaling, ultimately resulting in decreased p-AKT expression and increased phosphatase and tensin homolog (PTEN) expression. Therefore, RES induces apoptosis in OC cells via the ROS/Notch1/PTEN/AKT signaling pathway (Table 1; Figure 2).

**TABLE 1:** Effects of RES on the signaling pathways of apoptosis.

Study type	Cancer types	Models	Dosage/concentrations	Effects and signaling pathways (↑upregulation, ↓downregulation)	Ref
*in vitro*	CC	HeLa cells	0, 5, 10, 20, 40 µM	Bax↑; caspase-3 and -9↑; Bcl-2↓; Bcl-XL↓	Li et al. (2018)
	OC	A2780 cells	200 µL	Bax↑; caspase-3↑	Synowiec et al. (2023)
	OC	SKOV-3 and OV-90 cells	100 µM	miR-34a↑; Bcl-2↓	Yao et al. (2021)
	OC	SKOV-3 cells	20, 40, 80 μmol/L	Bax↑; caspase-3↑; Bcl-2↓	Tang et al. (2015)
	CC	HeLa and Ca Ski cells	10, 20, 40 µM	E6↓; E7↓; p53↑; Bax↑; G1/S-phase block↑	Sun et al. (2021a)
	CC	HeLa cells	5–50 µM	E6↓; p53↑	Chatterjee et al. (2018)
	CC	HeLa cells	40, 80 µM	p53↑; G1/S-phase block↑	Flores-Pérez and Elizondo (2018)
	OC	PA-1, MDAH2774 and SKOV3 cells	50 µM	p-AKT↓; p-GSK3β(S9) ↓; ENTPD5↓; NLG↓; GADD153↑; p-PERK↑; ATF6α↑	Gwak et al. (2016)
	CC	HeLa cells	60 μg/mL	NCLX↑; Ga^2+^↑	Devi et al. (2021)
	CC	HeLa cells	40, 80 μmol/L	FOXO3a↑; Bim↑; p-ERK↓; p-FOXO3a↓ and promote FOXO3a nuclear translocation	Liu et al. (2020)
	OC	A2780 and SKOV-3 cells	100 µM	ROS↑; Notch1↓; p-PTEN↑; P-AKT↓	Kim et al. (2019)

Bcl-2, B-cell lymphoma-2; Bax, Bcl-2-associated X; Bovine B-cell lymphoma-XL; E6, Human papillomavirus E6 protein; E7, Human papillomavirus E7 protein; p-AKT, phospho-Protein kinase B; p-GSK3β(S9), phospho-glycogen synthase kinase-3β(Ser9); ENTPD5, ER UDPase, ectonucleoside triphosphate diphosphohydrolase 5; NLG, N-linked glycosylation; GADD153, rowth arrest/DNA, damage-inducible protein153; p-PERK, phospho-Protein Kinase RNA-like Endoplasmic Reticulum Kinase; ATF6α, Recombinant Activating Transcription Factor 6-α; NCLX, sodium/lithium/calcium exchanger; FOXO3a, Forkhead box O3; Bim, Bim Protein; p-ERK; phospho-Extracellular Regulated Protein Kinases; FOXO3a, phospho-Forkhead box O3; ROS, reactive oxygen species; Notch1, Neurogenic locus notch homolog protein one; p-PTEN, phospho-Phosphatase and tensin homolog.

## 4 Role of RES in inhibiting tumor cell proliferation

Tumorigenesis is closely related to malignant tumor cell proliferation (Hanahan and Weinberg, 2011); therefore, inhibiting tumor cell proliferation presents an effective means of treating tumors.

### 4.1 Proliferating cell nuclear antigen (PCNA)

PCNA is a common marker of cell proliferation and plays a crucial role in processes, such as DNA replication, repair, and recombination (Cardano et al., 2020). In recent years, PCNA has emerged as a potential non-carcinogenic antitumor target, and its inhibition may control the abnormal proliferation of tumor cells (Kowalska et al., 2018; Horsfall et al., 2020). An *in vivo* experiment confirmed that by inhibiting PCNA protein expression, RES can block the cell cycle and inhibit proliferation, thereby significantly reducing the size of CC tumors (Chatterjee et al., 2019). Additionally, RES downregulated E6 and vascular endothelial growth factor expression. Zhang et al. (2020) reported that RES effectively inhibited NUTU-19 (OC cell line)-transplanted tumor growth in rats. Specifically, RES treatment significantly decreased G1/S-specific cyclin (Cyclin)-D1 and PCNA expression; notably increased PTEN, Fas ligand (FasL), and p21 expression; and inhibited the hepatocyte growth factor (HGF)/cellular-mesenchymal epithelial transition factor (c-Met) pathway.

### 4.2 Wnt signaling pathway

Wnt factors are secreted glycoproteins that play important roles in various biological processes involved in tumor growth and development (Cruciat and Niehrs, 2013). Targeting the Wnt signaling pathway can affect pathophysiological processes, such as cancer cell proliferation, apoptosis, and differentiation. Notably, the Wnt signaling pathway is crucial in ovarian carcinogenesis, development, invasion, and drug resistance (Nguyen et al., 2019). According to Wang and Shi (2019), RES modulates silent mating type information regulation two homolog (SIRT)-1 to inhibit OC cell proliferation and promote apoptosis through a mechanism likely related to the inactivation of the Wnt signaling pathway. Hou et al. (2020) demonstrated that RES significantly inhibits OC cell proliferation in a dose-dependent manner, an effect associated with Wnt signaling pathway activation suppression. Specifically, RES treatment decreased the mRNA expression levels of myelocytomatosis viral oncogene homolog (c-Myc), cyclin A, cyclin D1, N-cadherin, vimentin, p21, and E-cadherin as well as the protein expression levels of β-catenin and GSK3β.

### 4.3 Other mechanisms of RES-mediated inhibition of tumor cell proliferation

**FIGURE 3:**
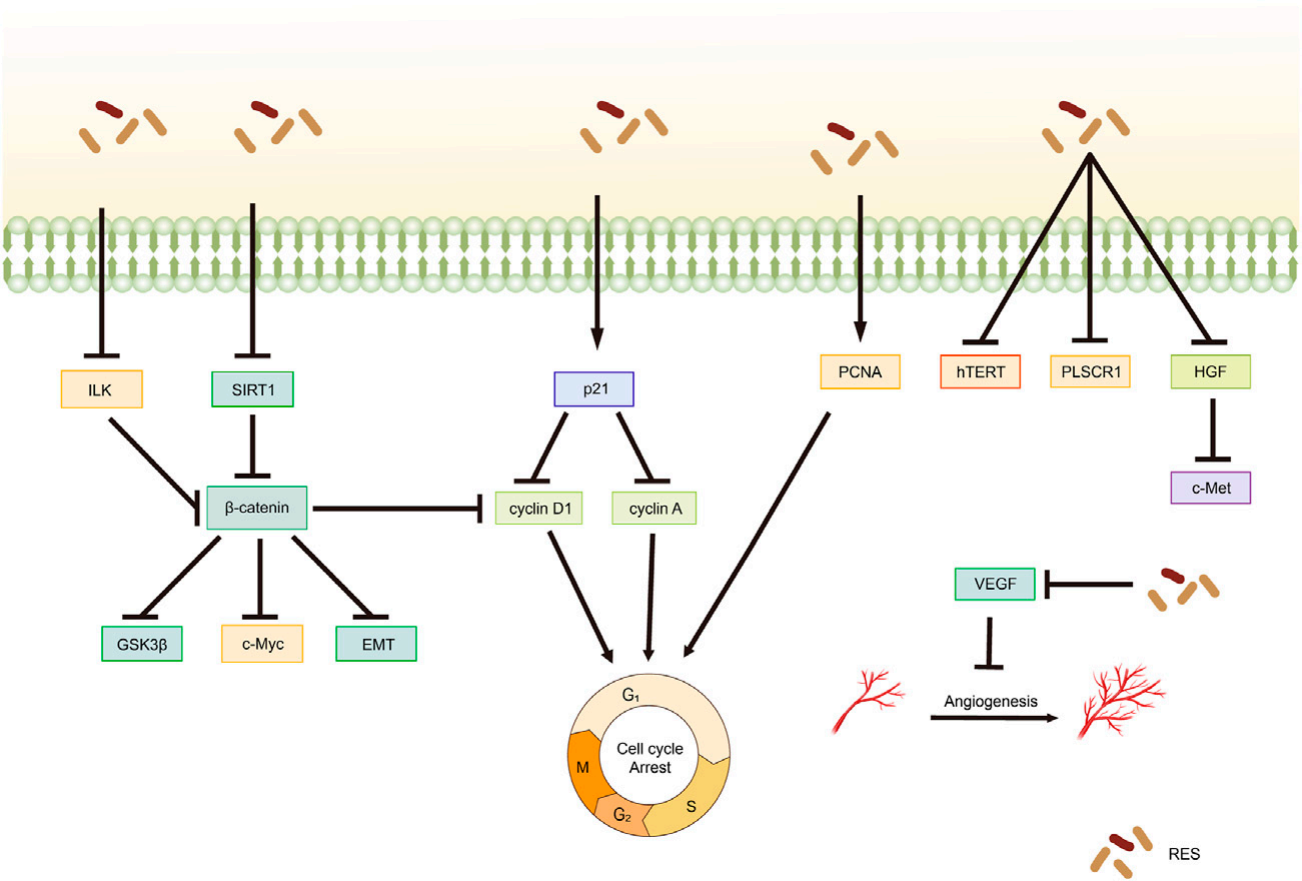
Mechanism of resveratrol suppressing proliferation in common gynecological tumors. RES, resveratrol; ILK, integrin-linked kinase; GSK3β, glycogen synthase kinase-3β; SIRT1, Silent mating type information regulation 2 homolog-1; c-Met, cellular-Mesenchymal epithelial transition factor; EMT, Epithelial–Mesenchymal transition; PCNA, proliferating cell nuclear antigen; hTERT, human telomerase reverse transcriptase; PLSCR1, Phospholipid scramblase 1; HGF, hepatocyte growth factor; c-Met, cellular-Mesenchymal epithelial transition factor; VEGF, Vascular endothelial growth factor.

Ma et al. (2021) demonstrated that RES inhibited the integrin-linked kinase/β-catenin pathway activity in A2780 cells, affecting the expression of the downstream target gene associated with cyclin D1, resulting in the inhibition of A2780 cell growth and proliferation. An *in vitro* experiment on CC revealed that telomerase activity was effectively inhibited by RES treatment in HeLa cells, with a concentration-dependent reduction in human telomerase reverse transcriptase (hTERT) expression levels being observed (He et al., 2019). This finding suggests that RES inhibits the telomerase activity of HeLa cells by regulating telomerase transcription, thereby inhibiting their proliferation. Furthermore, Zhao et al. (2019) reported that RES effectively inhibited CC cell proliferation by downregulating phospholipid scramblase one expression (Table 2; Figure 3).

**TABLE 2:** Effects of RES on the signaling pathways of proliferation.

Study type	Cancer types	Models	Dosage/concentrations	Effects and signaling pathways (↑upregulation, ↓downregulation)	Ref
*in vitro*	OC	A2780 cells	200 μmol/L	SIRT1↓; β-catenin↓; c-Myc↓	Wang and Shi (2019)
	OC	SKOV3 cells	20, 40, 80 μmol/L	c-Myc↓; cyclin A↓; cyclin D1↓; N-cadherin↓; Vimentin↓; β-catenin↓; p21↑; E-cadherin↑; GSK3β↑	Hou et al. (2020)
	OC	A2780 cells	200 μmol/L	ILK/β-catenin↓; cyclin D1↓; G0/G1-phase block↑	Ma et al. (2021)
	CC	HeLa cells	50, 100, 150 μmol/L	Effective inhibition of telomerase activity; hTERT↓	He et al. (2019)
*in vivo*	OC	NUTU-19 cells and Fischer 344 Rat ♀	50, 100, 200 mg/kg	Inhibits tumor growth in a dose-dependent manner; CyclinD1↓; PCNA↓; PTEN↑; FasL↑; p21 ↑; HGF/c-Met↓	Zhang et al. (2020a)
*in vitro* and *in vivo*	CC	TC1 cells; C57BL/6 mice♀	30 μM; 0.1 µM/uL	Significant reduction in tumor size; PCNA↓; VEGF↓	Chatterjee et al. (2019)
	CC	HeLa cells; BALB/C mice♂	1 μM; 10 mg/kg	PLSCR1↓; Inhibits tumor growth	Zhao et al. (2019)

SIRT1, Silent mating type information regulation two homolog-1; c-Myc, Myelocytomatosis viral oncogene homolog; N-cadherin, Neural-cadherin; GSK3β, glycogen synthase kinase-3β; ILK, integrin-linked kinase; hTERT, human telomerase reverse transcriptase; PCNA, proliferating cell nuclear antigen; PTEN, phosphatase and tensin homolog; FasL, fas ligand; HGF, hepatocyte growth factor; c-Met, cellular-Mesenchymal epithelial transition factor; VEGF, vascular endothelial growth factor; PLSCR1, Phospholipid scramblase 1.

## 5 Role of RES in inhibiting metastasis and tumor cell invasion

Migration and invasion of cancer cells are crucial factors in accelerating cancer progression, closely related to poor prognosis and causing death in patients with cancer (Padmanaban et al., 2019). Inhibition of tumor metastasis is an important measure for delaying tumor progression (Paul et al., 2017).

### 5.1 Epithelial–mesenchymal transition (EMT)

EMT is a critical step in the process of tumor migration and invasion. EMT can be induced or regulated by various growth and differentiation factors. Moreover, EMT is associated with the progression, invasion, and metastasis of a wide range of tumors (Kalluri, 2009; Koren et al., 2015; Wang et al., 2020b; Taki et al., 2021). Sun et al. (2020) demonstrated that RES suppresses the metastatic potential of CC *in vitro* and *in vivo* by inhibiting the phosphorylation of signal transduction and transcriptional activator 3 (STAT3) at the Tyr705 site. Specifically, RES downregulated the intracellular expression levels of N-cadherin, vimentin, matrix metalloproteinase (MMP)-3, and MMP-9 and increased the expression of E-cadherin, likely through EMT inhibition and extracellular matrix degradation. Li et al. (2020b) showed that RES effectively inhibited the invasion and migration of SKOV3 cells. After RES treatment with RES, the phosphorylation levels of interleukin (IL)-6, phospho-Janus kinase (JAK) 2, and STAT3 phosphorylation levels significantly decreased, suppressing the EMT process. This suggests that RES inhibits the invasive and migratory abilities of OC cells by downregulating the JAK2/STAT3 signaling pathway. Additionally, Kim et al. (2016) revealed that RES inhibits norepinephrine (NE)-induced OC cell invasiveness by inhibiting tyrosine protein kinase (Src) phosphorylation and hypoxia-inducible factor (HIF)-1α expression, thereby reducing NE-induced hTERT expression, inhibiting Slug expression, and ultimately blocking the NE-induced EMT process.

### 5.2 Other mechanisms of RES in suppressing tumor metastasis and invasion

**FIGURE 4:**
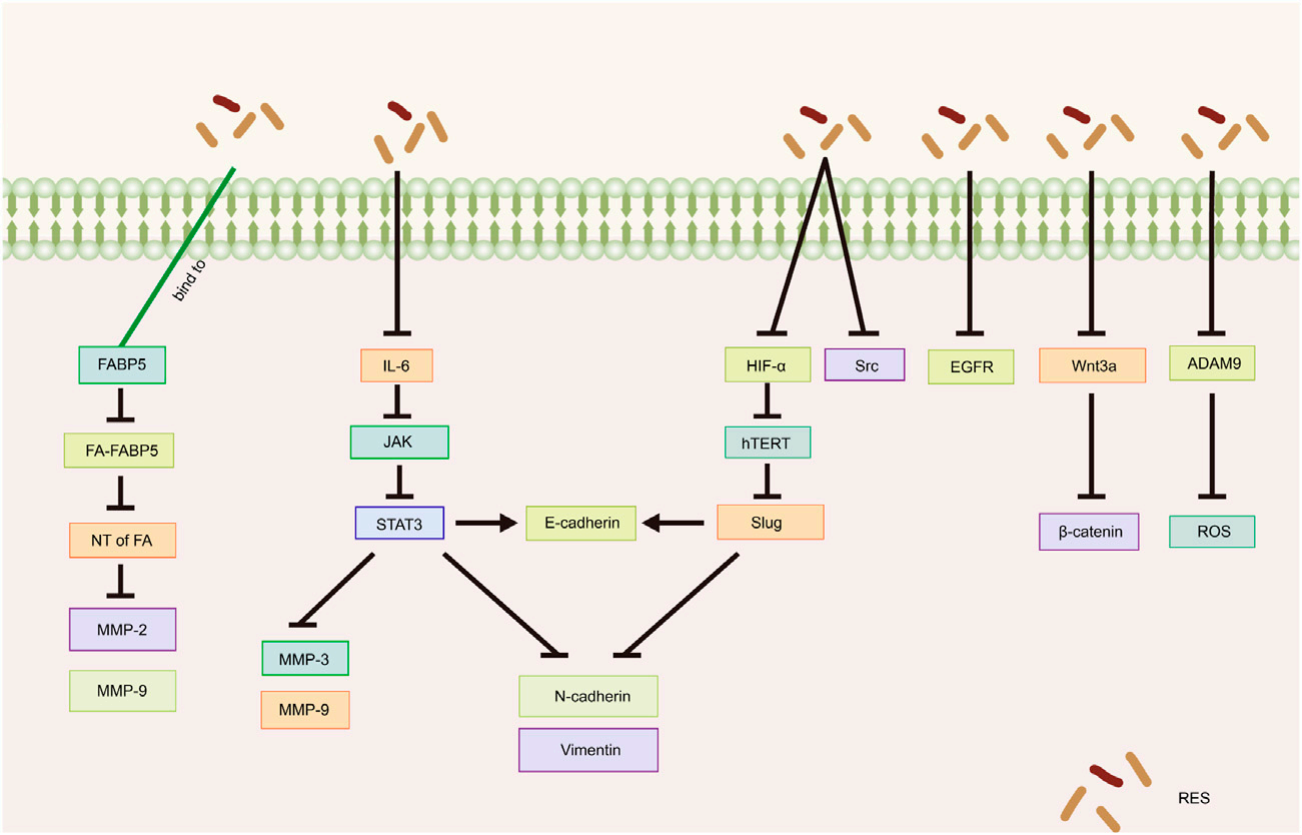
Mechanism of resveratrol suppressing metastasis and invasion in common gynecological tumors. RES, resveratrol; FABP5, Fatty Acid Binding Protein 5; FA, fatty acids; NT of FA, Nuclear transfer of fatty acids; MMP-2, Matrix metalloproteinase-2; MMP-9, Matrix metalloproteinase-9; IL-6, Interleukin-6; JAK, Janus kinase; STAT3, Signal transducer of activators of transcription 3; MMP-3, Matrix metalloproteinase-3; N-cadherin, Neural-cadherin; HIF-1α, Hypoxia Inducible Factor-1α; hTERT, human telomerase reverse transcriptase; Slug, SNAI2; Src, Tyrosine protein kinase; EGFR, Epidermal growth factor receptor; ADAM9, A disintegrin and metalloproteinase 9; ROS, reactive oxygen species.

Jeong et al. (2013) showed that RES attenuates lysophosphatidic acid-induced OC invasion by inhibiting epidermal growth factor receptor activation. According to Chen et al. (2023), RES effectively curtails the migration and metastasis of CC cells and improves survival rates in murine models. Specifically, by directly binding to fatty acid binding protein 5 (FABP5), RES competitively inhibits fatty acid binding to FABP5, consequently inhibiting MMP-2 and MMP-9 expression. Additionally, Li et al. (2019) showed that *in vitro*, RES inhibited the migration and invasion of SKOV3 cells, likely related to the inhibition of the Wnt3a/β-catenin axis. The disintegrin and metalloproteinase (ADAM) family is associated with various biological processes, including angiogenesis, cell–cell interactions, and migration (Schumacher et al., 2020). A recent study demonstrated that by inhibiting ADAM9 protein expression, RES reduces ROS production, significantly inhibiting the migratory ability of CC cells (Nan et al., 2021) (Table 3; Figure 4).

**TABLE 3:** Effects of RES on the signaling pathways of metastasis and invasion.

Study type	Cancer types	Models	Dosage/concentrations	Effects and signaling pathways (↑upregulation, ↓downregulation)	Ref
*in vitro*	OC	SKOV3 cells	1.0*10^6^, 5.0*10^6^, 2.50*10^5^ mol/L	p-IL-6↓; p-JAK2↓; p-STAT3↓; EMT↓	Li et al. (2020a)
	OC	SKOV3 and PA-1 cells	25 or 10 µM	p-Src↓; HIF-1α/hTERT/Slug↓	Kim et al. (2016)
	OC	SKOV3 and PA-1 cells	25 µM	p-EGFR↓	Jeong et al. (2013)
	OC	SKOV3 cells	20, 40 μg/mL	Wnt3a/β-catenin↓	Li et al. (2019)
	CC	HeLa cells	20 μmol/mL	ADAM9↓; ROS↓	Nan et al. (2021)
*in vitro* and *in vivo*	CC	HeLa and SiHa cells BALB/C mice♀	5, 10, 20, 40 µM 30 mg/kg	Inhibition of growth and metastatic potential of xenograft tumors; p-STAT3^Tyr705^↓; N-cadherin↓; Vimentin↓; MMP-3↓; MMP-9↓; E-cadherin↑	Sun et al. (2020)
	CC	HeLa and SiHa cells BALB/C mice♀	10, 20, 40, 60, 80 µM 25, 50, 100 mg/kg	Inhibition of xenograft tumor metastasis in a concentration-dependent manner; Inhibits fatty acid transport to the nucleus by competitively inhibiting fatty acid binding to FABP5; MMP-2↓; MMP-9↓	Chen et al. (2023)

p-IL-6, phospho-Interleukin-6; p-JAK2, phospho-Janus kinase two; p-STAT3, phospho-Signal transducer of activators of transcription 3; EMT, Epithelial-mesenchymal transition; p-Src, phospho-Tyrosine protein kinase; HIF-1α, Hypoxia Inducible Factor-1α; hTERT, human telomerase reverse transcriptase; Slug, SNAI2; p-EGFR, phospho-Epidermal growth factor receptor; ADAM9, A disintegrin and metalloproteinase 9; ROS, reactive oxygen species; N-cadherin, Neural-cadherin; MMP-3, Matrix metalloproteinase-3; MMP-9, Matrix metalloproteinase-9; FABP5, Fatty Acid Binding Protein 5; MMP-2, Matrix metalloproteinase-2.

## 6 Role of RES in autophagy regulation in tumor cells

Cellular autophagy is an intracellular self-degradation process that maintains normal cellular homeostasis by lysosomal hydrolysis of excessive or damaged organelles and pathogens (Li et al., 2020c). Autophagy can exhibit a dual role, acting either via an antitumor or a protumor mechanism, depending on the stage of cancer development (Zhang Y. et al., 2020).

**FIGURE 5:**
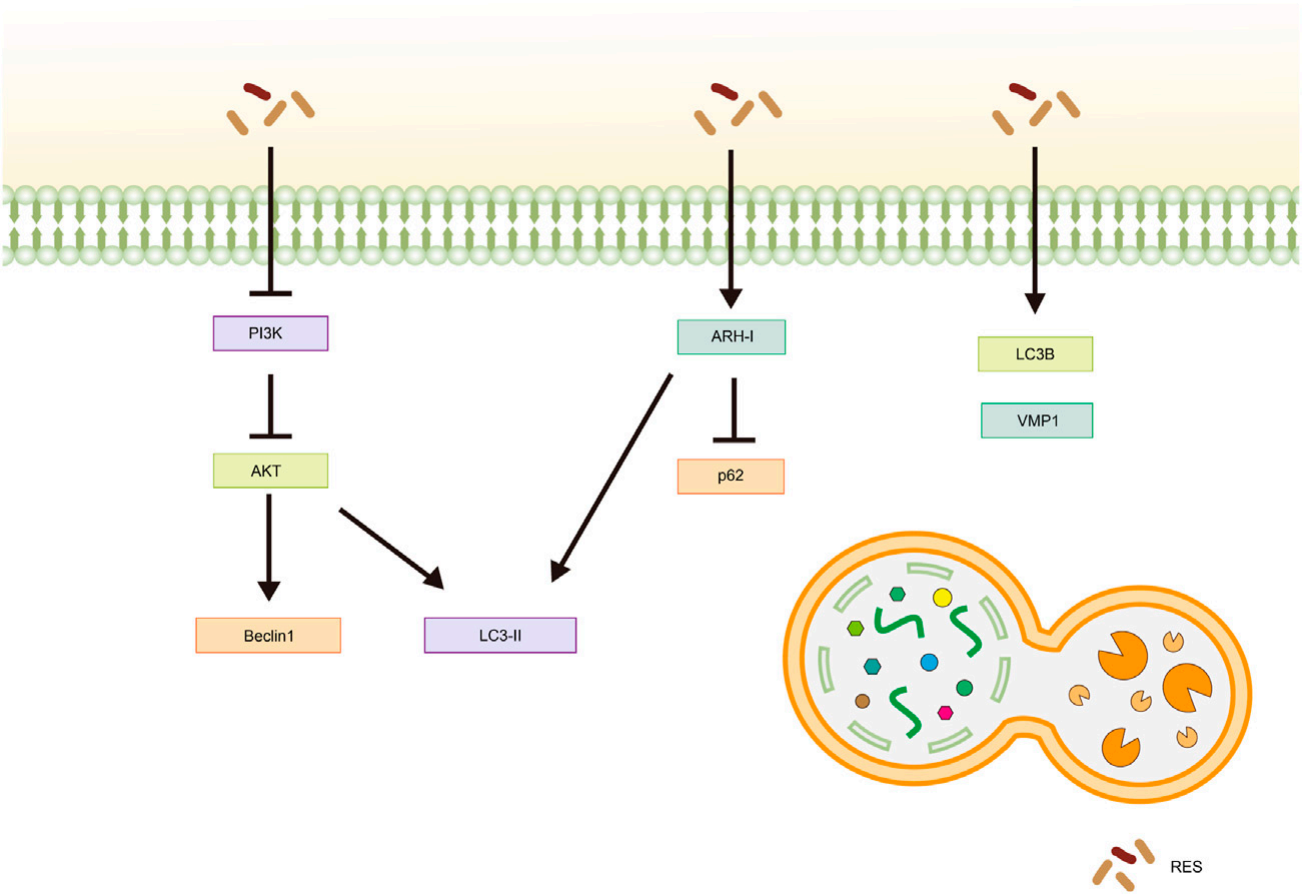
Mechanism of resveratrol inducing autophagy in common gynecological tumors. RES, resveratrol; PI3K, phosphatidylinositol 3-kinase; AKT, Protein Kinase B; LC3-Ⅱ, Microtubule-Associated Protein 1 Light Chain 3-Ⅱ; ARH-I, Aplysia Ras Homolog-I; LC3B, Microtubule-Associated Protein 1 Light Chain 3B; VMP1, vacuole membrane protein-1.

Fan et al. (2022) observed increased expression of autophagy-related gene six homolog 1 (Beclin1) and microtubule-associated protein one light chain 3 (LC3)-Ⅱ, as well as a rise in the number of autophagic vesicles in cells following RES treatment. Further studies have shown that RES promotes autophagy in Ishikawa cells by inhibiting the phosphatidylinositol 3 kinase (PI3K)/AKT signaling pathway, thereby inhibiting the malignant proliferation of EC (Fan et al., 2022). Aplysia ras homolog I (ARH-I) regulates cellular autophagy (Yan et al., 2019) and is expressed at low levels in OC cells (Washington et al., 2015). In a 3D sphere culture model of OC cell lines (OVCAR3, OAW42, and KURAMOCHI), Esposito et al. (2022) found that IL-6 inhibited autophagy by downregulating ARH-I expression via miR-1305. In contrast, ARH-Ⅰ and LC3-Ⅱ increased, p62 levels decreased, and autophagy was activated in tumor spheroids following RES treatment. Moreover, according to Wang et al. (2019), RES treatment resulted in increased expression levels of the autophagy-related proteins LC3-Ⅱ, Beclin-1, and vacuole membrane protein-1 in SKOV-3 cells, highlighting its potential for inducing autophagy in tumor cells. In another study, Sun X. D. et al. (2021) showed that RES significantly promoted LC3B and Beclin-1 expression and inhibited P62 expression in tumor tissues *in vivo* and *in vitro*, hypothesizing that RES inhibits CC by promoting autophagic death in HeLa cells. Sun et al. (2018) also reported similar results in an *in vitro* assay using HeLa cells (Table 4; Figure 5).

**TABLE 4:** Effect of RES on the signaling pathways of autophagy.

Study type	Cancer types	Models	Dosage/concentrations	Effects and signaling pathways (↑upregulation, ↓downregulation)	Ref
*in vitro*	EC	Ishikawa cells	100 μmol/L	Beclin1↑; LC3-Ⅱ↑; Number of autophagic vesicles↑; PI3K/AKT↓	Fan et al. (2022)
	OC	OVCAR3, OAW42, and KURAMOCHI cells	10 µM	ARH-I↑; LC3-Ⅱ↑; p62↓	Esposito et al. (2022)
	OC	SKOV3 cells	25 μmol/L	LC3-Ⅱ↑; Beclin-1↑	Wang and Shi (2019)
	CC	HeLa cells	0–100 μmol/L	LC3↑; Beclin-1↑; p62↓	Sun et al. (2018)
*in vitro* and *in vivo*	CC	HeLa and SiHa cells BALB/C mice♀	10, 20, 40 µM 50, 100 mg/kg	LC3B↑; Beclin-1↑; VMP1↑; p62↓; ∆Ψm↓	Sun et al. (2021a)

LC3, Microtubule-associated protein one light chain 3; PI3K, phosphatidylinositol 3-kinase; AKT, Protein Kinase B; ARH-I, Aplysia Ras Homolog-I; LC3-Ⅱ, Microtubule-Associated Protein 1 Light Chain 3-Ⅱ; LC3B, Microtubule-Associated Protein 1 Light Chain 3B; VMP1, vacuole membrane protein-1; ∆Ψm, Mitochondrial membrane potential.

## 7 Combined with antitumor drugs, RES increases efficacy and reduces toxic side effects

Chemotherapy remains a cornerstone in tumor treatment despite the initial responsiveness of patients with tumors. However, most patients develop acquired resistance after long-term treatment, which is one of the main reasons for unsatisfactory tumor treatment and disease recurrence (Zhang et al., 2022). Consequently, identifying chemotherapeutic sensitizers to prevent and reverse multidrug resistance in tumor cells has become a pressing priority. RES acts synergistically with existing antitumor drugs by enhancing the sensitivity of cancer cells to chemotherapeutic drugs, effectively reversing multidrug resistance and reducing the toxic side effects during chemotherapy. The synergistic effect of RES and other anticancer drugs involves the regulation of multiple signaling pathways and cellular processes, including activation of p53, reduction of the expression of P-glycoprotein (P-gp), NF-κB, and TNF-α expression, and inhibition of the PI3K/AKT/mammalian rapamycin target protein (mTOR) axis. With these synergistic effects, RES can significantly enhance the impact of anticancer drugs, providing new strategies for cancer treatment.

### 7.1 Potentiating effect of RES on antitumor drugs

#### 7.1.1 Cisplatin (CDDP)

CDDP is considered a first-line therapeutic agent due to its broad-spectrum antitumor effects and significant results in clinical results. However, most patients develop inherent or acquired drug resistance to CDDP (Rocha et al., 2018). RES treatment significantly enhances the sensitivity of SKOV-3 cells to CDDP (Hankittichai et al., 2023). Specifically, the combination of RES and CDDP activates caspase-3, caspase-9, and poly (ADP-ribose) polymerase (PARP); inhibits cyclin A2 and cyclin B1 expression; and induces cell cycle arrest in the S phase, ultimately inhibiting cell proliferation and promoting apoptosis. Further experiments revealed that RES strongly activates p38 phosphorylation and inhibits AKT activation (Hankittichai et al., 2023). Yue et al. (2019) showed that RES enhances the sensitivity of HeLa cells to CDDP by inducing G1 phase blockage and apoptosis. Mechanistically, p21, Bax, caspase-3, and caspase-9 expression increased, whereas cyclin-dependent kinase 2 (CDK2), cyclin D1, and Bcl-2 expression decreased in cells treated with the combination treatment group compared to those treated with CDDP alone. Muhanmode et al. (2022) showed that RES and curcumin reversed OC-acquired CDDP resistance *in vitro* by significantly inhibiting the PI3K/AKT/mTOR axis. Jiang et al. (2022) demonstrated that RES treatment significantly enhances the sensitivity of SiHa cells to CDDP by activating SIRT3. Specifically, combination treatment increases the levels of hydrogen peroxide (H2O2) and manganese superoxide dismutase (MnSOD) in the cells and significantly reduces mitochondrial membrane potential (ΔΨm) and ROS levels. P-gp, a membrane transporter crucial for recognizing and expelling chemotherapeutic agents, reduces their intracellular accumulation and efficacy (Robey et al., 2018; Afonso de Lima et al., 2020). Wang et al. (2021) showed that RES reduces P-gp expression and reversed CDDP resistance in OC (SKOV-3) cells by downregulating multidrug resistance protein (MDR)-1 and Bcl-2 mRNA expression.

Hypoxia is a major hallmark of any solid tumor. Synowiec et al. (2023) demonstrated that the combination of RES and CDDP was ineffective in a hypoxic environment and that RES counteracted the pharmacological effects of CDDP in OC (A2780 cells). This highlights the need to consider factors, such as hypoxic status, when evaluating the impact of RES on CDDP chemosensitization and conducting assays under conditions mimicking the tumor microenvironment (TME).

#### 7.1.2 Additional potentiation of RES

Docetaxel is a paclitaxel-based antitumor drug with significant effects on advanced malignant tumors. The experimental results of Dou et al. (2022) in OC rats with loaded tumors showed that RES enhanced the inhibitory effect of docetaxel on the growth of xenograft tumor growth, suggesting that the mechanism may be related to an increase in Bax and caspase-3 expression and inhibition of Bcl-2 expression. Doxorubicin (DOX) is widely used as a chemotherapeutic agent for treating various hematological and solid cancers, including osteosarcoma, cervical cancer, and breast cancer. A study on OC stem cells confirmed that RES enhances the sensitivity of SKOV3 cells to DOX by activating the mitochondrial apoptotic pathway and inhibiting MDR-1 and multidrug resistance-associated protein-1 expression (Pouyafar et al., 2019) (Table 5).

**TABLE 5:** Potentiating effect of RES on antitumor drugs.

Study type	Standard anti-cancer drugs	Cancer types	Models	Dosage/concentrations	Effects and signaling pathways (↑upregulation, ↓downregulation)	Ref
*in vitro*	CDDP	OC	SKOV3 cells	35, 50, 100 µM	p-p38↑; AKT↓; cyclin A2↓; cyclin B1↓; caspase-3↑; caspase-9↑; PARP↑; S-phase block↑	Hankittichai et al. (2023)
		CC	HeLa and HeLa/CDDP cells	4 μg/mL	p21↑; Bax↑; caspase-3↑; caspase-9↑; CDK2↓; cyclin D1 ↓; Bcl-2↓; G1-phase block↑	Yue et al. (2019)
		OC	A2780 and A2780-cis cells	70 µM	PI3K/AKT/mTOR↓	Muhanmode et al. (2022)
		CC	SiHa cells	5 µM	SIRT3↑; MnSOD↑; ROS↓; H_2_O_2_↑; ∆Ψm↓	Jiang et al. (2022)
		OC	SKOV3/CDDP cells	100 U/mL	MDR-1↓; Bcl-2↓; P-gp↓	Wang et al. (2021)
	DOX	OC	SKOV3 cells	55 µM	Bax↑; Caspase-3↑; Bcl-2↓; MDR-1↓; MRP-1↓	Pouyafar et al. (2019)
*in vivo*	Docetaxel	OC	NUTU-19 cells and Fischer 344 Rat ♀	200 mg/kg	Inhibition of xenograft tumor growth; Bax↑; Caspase-3↑; Bcl-2↓	Dou et al. (2022)

CDDP, Cisplatin; DOX, Doxorubicin; p-p38, phospho-p38; AKT, Protein kinase B; PARP, poly (ADP-ribose) polymerase; Bax, Bcl-2-associated X; CDK2, cyclin-dependent kinase 2; Bcl-2, B-cell lymphoma-2; PI3K, Phosphatidylinositol-3-kinase; mTOR, Mammalian rapamycin target protein; SIRT3, Silent mating type information regulation two homolog-3; MnSOD, Manganese Superoxide Dismutase; ROS, reactive oxygen species; H_2_O_2_, hydrogen peroxide; ∆Ψm, Mitochondrial membrane potential; MDR-1, Multidrug Resistance Protein 1; P-gp, P-glycoprotein; MRP-1, Multidrug Resistance-associated Protein 1.

### 7.2 RES attenuates damage to the reproductive system caused by antitumor drugs

Chemotherapeutic agents can damage the female reproductive system, with ovaries being one of the most commonly affected organs (Morgan et al., 2012).

#### 7.2.1 CDDP

CDDP use may result in ovarian hypoplasia or exhaustion (Meirow, 2000; Rossi et al., 2017). Ciplak et al. (2023) showed that RES prevents and attenuates CDDP toxicity in ovarian follicles by increasing superoxide dismutase and catalase levels and decreasing malondialdehyde levels in OC tissue. Similar results were obtained by Chinwe et al. (2018), who hypothesized that RES promotes follicle-stimulating hormone expression. Said et al. (2019) noted that RES, by decreasing PARP1 expression, reversed the CDDP treatment-induced increase in the expression of the inflammatory factors cytochrome c and caspase-3. Another study showed that RES coadministration ameliorated CDDP-mediated ovarian and uterine damage in rats by decreasing the expression of inflammatory factors (NF-κB, tumor necrosis factor-α, cyclooxygenase-2), oxidative stress markers, and increasing cellular estradiol, progesterone, and prolactin levels (Ibrahim et al., 2021). However, in this study, the coadministration of RES and CDDP downregulated follicle-stimulating hormone expression.

#### 7.2.2 Other attenuative effects of RES

Wu et al. (2019) reported that RES effectively alleviates chemotherapy-induced ovarian stress and attenuates oogonial stem cell damage via SIRT1/FOXO1 axis activation. H_2_O_2_ was further used to establish an oxidative stress injury model. The results showed that nuclear factor erythroid-2 related factor (Nrf) 2 and superoxide dismutase 2 (SOD2) levels increased in RES-treated cells, and H_2_O_2_-induced apoptosis was significantly reduced. Herrero et al. (2023) found that RES effectively attenuated DOX-induced ovarian injury in a murine model, with the mechanism related to the protection of primordial follicular cells, promotion of follicular cell proliferation, and inhibition of DOX-mediated changes in endogenous apoptosis (Table 6).

**TABLE 6:** RES attenuates damage to the reproductive system caused by antitumor drugs.

Study type	Standard anti-cancer drugs	Models	Dosage/concentrations	Effects and signaling pathways (↑upregulation, ↓downregulation)	Ref
*in vivo*	CDDP	Wistar-Albino rat♀	10 mg/kg	Protection against CDDP-induced oxidative stress-induced ovarian damage; MDA↓; SOD↑; CAT↑	Ciplak et al. (2023)
		Sprague-Dawley rat♀	5, 10, 20 mg/kg	Protection against CDDP-induced oxidative stress-induced ovarian damage; SOD↑; CAT↑; GSH↑; MDA↓; progesterone↑; LH↑; FSH↑	Chinwe et al. (2018)
		Sprague–Dawley rat♀	10 mg/kg	Alleviation of CDDP-induced ovarian toxicity; NF-κB↓; TNF-α↓; COX-2↓; iNOS↓; Cyt-c↓; capase-3↓; PARP1↓	Said et al. (2019)
		Wistar rats♀	10 mg/kg	Attenuation of CDDP-induced ovarian and uterine toxicity; PARP1↓; NF-kB↓; TNF-α↓; COX-2↓; IL-1β↓; IL-6↓; Cyt-c↓; capase-3↓; GSH↑; SOD↑; CAT↑; GPx↑	Ibrahim et al. (2021)
	DOX	First generation mice produced by crossing C57BL/6 mice♂ with BALB/C mice♀	7, 15 mg/kg	Alleviating chemotherapy-induced ovarian stress response; protection of primordial follicular cells, promotion of follicular cell proliferation; inhibition of DOX-mediated changes in endogenous apoptosis	Herrero et al. (2023)
*in vitro* and *in vivo*	Bu/Cy	C57BL/6 mice♀; OSCs isolated from C57BL/6 mice♀	30 mg/kg; 2, 5 µM	Alleviating chemotherapy-induced ovarian stress response; Attenuating OSCs injury; SIRT1/FOXO1↑; Nrf2↑; SOD2↑	Wu et al. (2019)

CDDP, cisplatin; MDA, malondialdehyde; SOD, superoxide dismutase; CAT, catalase; GSH, glutathione; LH, luteinizing hormone; FSH, follicle-stimulating hormone; NF-κB, nuclear factor kappa B; TNF-α, tumor necrosis factor-α; COX-2, Cyclooxygenase-2; iNOS, inducible nitric oxide synthase; Cyt-c, Cytochrome c; PARP1, poly (ADP-ribose) polymerase 1; IL-1β, interleukin-1β; IL-6, interleukin-6; GPx, glutathione peroxidase; DOX, doxorubicin; Bu/Cy, busulfan/cyclophosphamide; OSCs, Ovarian Stem Cells; SIRT1, Silent mating type information regulation two homolog-1; FOXO1, Forkhead box protein O1; Nrf2, Nuclear Factor Erythroid-2, Related Factor 2; SOD2, Superoxide dismutase 2.

## 8 RES nanoparticles for improved bioavailability

Due to the poor water solubility, rapid metabolism, and limited bioavailability of RES, establishing suitable carriers to enhance its delivery is imperative (Asensi et al., 2002; Walle, 2011; Sharifi-Rad et al., 2021). A nanodelivery system can compensate for these shortcomings of the drug itself without altering the active ingredient, improving bioavailability and stability, and achieving targeted drug delivery (Cheng et al., 2008; Pelaz et al., 2017; Li et al., 2020). Long et al. (Long et al., 2020) used RES, arginine-glycine aspartic acid, and human serum albumin to prepare a novel nanocomplex, which exerted an inhibitory effect on SKOV3 cells in a time- and dose-dependent manner *in vitro*. Moreover, RES significantly inhibited tumor growth in OC model mice *in vivo* (Long et al., 2020). Tomoaia et al. (2015) developed gold nanoparticles (GNPs) modified with RES that were used to construct nanocomplexes of DOX-GNPs. These GNPs demonstrated a strong accumulation effect and inhibited CC progression through apoptosis induction, showing greater effectiveness in CaSki cells than in HeLa cells.

## 9 Metabolic, hormonal, and immunological effects of RES in cancer

### 9.1 Glycolysis

Metabolic reprogramming is a key indicator of cancer development and progression. Glycometabolism is a major cellular energy production pathway. Glycolysis, a basic process of gluconeogenesis, plays a crucial role in cellular metabolism, and its enhancement is a prominent feature of cancer metabolic processes (Liberti and Locasale, 2016; Zhong et al., 2018; Wu et al., 2020). Therefore, inhibiting the glycolytic pathway inhibition should be considered in tumor treatment. Vidoni et al. (2023) demonstrated that RES treatment significantly inhibits glucose uptake and glycolysis, decreases ROS expression, promotes cellular autophagy, and inhibits cancer cell metastasis in OC cells (OVCAR3 and OAW42). Tan et al. (2016) experimentally demonstrated that RES treatment inhibited glycolysis in A2780 cells and suppressed the growth of murine xenograft tumors. Another study confirmed that the anti-OC effects of RES were achieved by inhibiting glycolysis. Moreover, RES induced activation of the AMPK signaling pathway, inhibited the expression and activation of mTOR, increased the apoptosis level, and inhibited the proliferation, migration, and invasion of A2780 and SKOV3 cells, exerting its antitumor effects (Liu et al., 2018). Further *in vivo* investigations revealed that RES treatment effectively inhibited the growth and metastasis of murine xenograft tumors. Gwak et al. (2015) demonstrated that via AKT activity, RES suppressed the translocation of glucose transporter one and further reduced the glucose uptake capacity of OC cells (PA-1, OVCAR3, MDAH2774, and SKOV3), thus exerting antitumor effects.

### 9.2 Estrogenic effects

EC is a common gynecological malignancy closely related to estrogen levels (Rodriguez et al., 2019). When estrogen binds to estrogen receptors on the surface of tumor cells, it initiates various signaling pathways that play important roles in the formation, infiltration, and metastasis of EC (Hawsawi et al., 2013; Medhin et al., 2020). Various estrogen metabolites may cause genotoxic damage (Cavalieri et al., 2006). For example, 4-hydroxyestradiol (4-OHE2) and 2-methoxyestradiol (2-MeOE2) are major carcinogens among estrogen metabolites and are considered tumor markers in patients with EC (Zhao et al., 2015). These metabolites bind to DNA via a non-receptor-mediated pathway to form adducts that trigger DNA-coding errors, induce DNA damage, and promote cancer development (Saeed et al., 2009). Therefore, the occurrence and development of EC are closely associated with the abnormal accumulation of estrogen and its toxic metabolites.

Notably, RES acts as a phytoestrogen that can bind to estrogen receptors and exert estrogen-like effects (Gehm et al., 1997; Sato et al., 2003). RES-induced apoptosis in breast cancer MCF-7 cells can be inhibited by E2 (Zhang et al., 2004). A recent study revealed that RES modulated estrogenic homeostasis in mice with endometrial cancer (Yin et al., 2019). Using a murine model of EC induced by N-methyl- N′-nitro-N-nitrosoguanidine, Yin et al. (2019) found that RES effectively decreased 4-OHE2 levels and increased 2-MeOE2 levels, thereby inhibiting EC development and progression.

### 9.3 Regulation of immunity

Immune surveillance plays a crucial role in recognizing and destroying cancer cells. In the TME, the function of immune cells is significantly suppressed, providing a protective mechanism for tumor cells to successfully achieve immune escape. Notably, the suppressive effect of immune cells in the TME is a critical step in the initiation of tumor metastasis before its occurrence (Liu and Cao, 2016). Therefore, enhancing immunity has been established as a core strategy for cancer prevention and treatment. According to Chen et al. (2022), RES reduces pyruvate kinase isozyme type M2 (PKM2) and glucose transporter (GLUT) one expression, inhibiting the glycolytic process and reducing lactate production in OC cells, thereby reversing T-regulatory cell-mediated immunosuppression. Zhang et al. (2019) found that RES exerted antitumor effects on OC *in vitro* and *in vivo* by inducing immunogenic cell death. Further studies showed that in loaded mice, RES improved the TME, promoted IL12p7 and IFN-γ production, inhibited transforming growth factor (TGF)-β expression, and significantly increased the number of CD80^+^, CD86^+^, and CD8 cells. Additionally, the experimental results of Chen et al. (2022) and Zhang et al. (2019) demonstrated that RES, in combination with PD-1 antibody, significantly inhibited xenograft tumor growth.

## 10 Challenges, limitations, and prospects

RES exhibits promising antitumor activity in both *in vitro* and *in vivo* experiments; however, it still faces many challenges in practical applications. Due to the complexity and large number of cellular processes involved in carcinogenesis, the specific mechanism of the role of RES in the intricate cell signaling network requires further exploration. Additionally, the lipophilic nature and rapid metabolism of RES result in low bioavailability (Boocock et al., 2007a; Ren et al., 2021). Improving the bioavailability of RES in the human body is essential. Moreover, previous clinical studies have shown certain side effects, such as gastrointestinal reactions (including nausea, flatulence, and diarrhea) and kidney injury (la Porte et al., 2010; Popat et al., 2013). Therefore, an in-depth investigation of the possible side effects and related safety issues associated with the long-term use of RES is required.

While RES demonstrates inhibitory effects on tumor cells under laboratory conditions, clinical trials remain integral to any drug development process (Patel et al., 2010). However, studies on the anticancer mechanisms of RES in common gynecological tumors have focused on rodent models and *in vitro* cellular experiments, with a paucity of clinical trial data. Although nearly 200 clinical studies have been conducted on RES, studies focusing on cancer treatment remain in the minority. Notably, no clinical trials have been documented on RES in the treatment of EC, CC, and OC (Brown et al., 2024). Accordingly, our understanding of the efficacy and safety of RES in actual patient populations remains limited. The doses of RES used in different studies vary considerably, and the lack of a uniform standardized dose increases the uncertainty of its clinical application. Furthermore, combining RES with nanocarriers can achieve precise targeted drug delivery, significantly enhancing drug bioavailability and reducing side effects. However, there is still a paucity of current research on RES nanoformulations specifically targeting common gynecological tumors.

Despite the many challenges and limitations, RES remains promising as a natural antitumor drug candidate. Future studies should focus on i) clinically validating the therapeutic efficacy of RES in common gynecological tumors through large-sample, high-quality randomized controlled double-blind trials to ensure the certainty of its efficacy; ii) developing nanoformulations of RES to enhance the bioavailability; and iii) using modern technical means to analyze and explore the anti-gynecological tumor mechanisms and mechanisms of action, providing more credible data to support for the development of new clinical drugs.

## 11 Conclusion

Gynecological cancers, which represent a significant health threat to women, have limited therapeutic options and present challenges in radical treatment. Due to the characteristics of malignant tumors, such as recurrence, distant metastasis, and chemotherapeutic drug resistance, the prognosis of such tumors is often poor, rendering the treatment process complicated and challenging. Therefore, there is an urgent need for comprehensive molecular-level investigations into their pathogenesis, and the active pursuit of efficient and low-toxicity therapeutic targets is urgently needed.

RES is a naturally occurring compound that exerts potent pharmacological effects, particularly in antitumor therapy. RES exhibits broad-spectrum antitumor effects and is effective against various cancer types, such as breast (Sun et al., 2019), pancreatic (Xiao et al., 2020), lung (Gao and Ren, 2021), gastric (Yin et al., 2020), and prostate cancers (Hsieh and Wu, 2020). RES exerts significant antitumor effects on common gynecological tumors via multiple mechanisms. Specifically, RES can induce apoptosis of tumor cells, inhibit cell proliferation, invasion, and metastasis, and regulate tumor cell autophagy. Additionally, RES improves the effectiveness, reduces the toxic side effects of anticancer drugs, and effectively inhibits the glycolytic process of tumor cells. RES can also regulate the immune microenvironment, which is closely related to tumors, and affect estrogen levels. At the molecular level, RES can promote cancer cell death by regulating the Bax/Bcl-2 ratio and the expression of key proteins, such as GSK3β and p53. Furthermore, RES further exerts its antitumor effects by regulating cell signaling pathways, such as miR-34a/Bcl-2, HGF/c-Met, ILK/β-catenin, and PI3K/AKT/mTOR.

In summary, RES, especially the use of RES-modified nanoparticles, shows remarkable potential for application in the treatment of common gynecological tumors. Further scientific and rational experimental studies and clinical validation are needed to explore the specific targets of RES and elucidate its mechanisms of action.

## Figures and Tables

**Table:** 

**RES**	Resveratrol
**CC**	Cervical carcinoma
**EC**	Endometrial carcinoma
**OC**	Ovary carcinoma
**Bcl-2**	B-cell lymphoma-2
**Bax**	Bcl-2-associated X
**Caspase-3**	Cysteine-requiring Aspartate Protease3
**E6**	Human papillomavirus E6 protein
**E7**	Human papillomavirus E7 protein
**UPR**	unfolded protein response
**AKT**	protein kinase B
**GSK3β**	glycogen synthase kinase-3β
**ENTPD5**	Ectonucleoside triphosphate diphosphohydrolase 5
**GADD153**	rowth arrest/DNA damage-inducible protein153
**NCLX**	sodium/lithium/calcium exchanger
**ERK**	extracellular signal-regulated kinase
**FOXO3a**	Forkhead box O3
**Bim**	Bim Protein
**ROS**	reactive oxygen species
**Notch1**	Neurogenic locus notch homolog protein 1
**PTEN**	Phosphatase and tensin homolog
**PCNA**	Proliferating Cell Nuclear Antigen
**VEGF**	vascular endothelial growth factor
**CyclinD1**	G1/S-specific cyclin-D1
**FasL**	Fas ligand
**HGF**	hepatocyte growth factor
**c-Met**	cellular-Mesenchymal epithelial transition factor
**SIRT1**	Silent mating type information regulation 2 homolog-1
**c-Myc**	Myelocytomatosis viral oncogene homolog
**N-cadherin**	Neural-cadherin
**ILK**	integrin-linked kinase
**hTERT**	human telomerase reverse transcriptase
**PLSCR1**	Phospholipid scramblase 1
**EMT**	Epithelial-Mesenchymal Transition
**STAT3**	signal transduction and transcriptional activator 3
**MMP-3**	Matrix metalloproteinase-3
**MMP-9**	Matrix metalloproteinase-9
**IL-6**	Interleukin-6
**JAK2**	phospho-Janus kinase 2
**NE**	norepinephrine
**Src**	Tyrosine protein kinase
**HIF-1α**	Hypoxia Inducible Factor-1α
**EGFR**	epidermal growth factor receptor
**FABP5**	Fatty Acid Binding Protein 5
**MMP-2**	Matrix metalloproteinase-2
**ADAM**	A disintegrin and metalloproteinase
**Beclin1**	Autophagy Related Gene 6 Homolog 1
**LC3-Ⅱ**	Microtubule-Associated Protein 1 Light Chain 3-Ⅱ
**PI3K**	phosphatidylinositol 3 kinase
**ARH-I**	aplysia ras homolog-I
**LC3B**	Microtubule-Associated Protein 1 Light Chain 3B
**VMP1**	vacuole membrane protein-1
**CDDP**	Cisplatin
**PARP**	poly (ADP-ribose) polymerase
**CDK2**	cyclin-dependent kinase 2
**mTOR**	Mammalian rapamycin target protein
**SIRT3**	Silent mating type information regulation 2 homolog-3
**MnSOD**	Manganese Superoxide Dismutase
**H2O2**	Hydrogen peroxide
**∆Ψm**	Mitochondrial membrane potential
**MDR-1**	Multidrug Resistance Protein 1
**P-gp**	P-glycoprotein
**DOX**	Doxorubicin
**MRP-1**	Multidrug Resistance-associated Protein 1
**SOD**	Superoxide dismutase
**CAT**	Catalase
**MDA**	Malondialdehyde
**FSH**	follicle stimulating hormone
**NF-κB**	nuclear factor kappa B
**TNF-α**	tumor necrosis factor-α
**COX-2**	Cyclooxygenase-2
**FOXO1**	Forkhead box protein O1
**Nrf2**	Nuclear Factor Erythroid-2 Related Factor 2
**AMPK**	Adenosine 5′-monophosphate (AMP)-activated protein kinase
**4-OHE2**	4-Hydroxyestradiol
**2-MeOE2**	2-methoxyestradiol
**TME**	Tumor microenvironment
**PKM2**	Pyruvate kinase isozyme typeM2
**GLUT1**	Glucose transporter-1
**IL12p7**	Interleukin-12 p7
**IFN-γ**	Interferon-γ
**TGF-β**	Transforming Growth Factor-β
